# Sensitization of Resistance Ovarian Cancer Cells to Cisplatin by Biogenic Synthesized Silver Nanoparticles through p53 Activation

**Published:** 2019

**Authors:** Tayebe Ramezani, Mohamad Nabiuni, Javad Baharara, Kazem Parivar, Farideh Namvar

**Affiliations:** a *Department of Animal Biology, Faculty of Biological Sciences, Kharazmi University, Tehran, Iran.*; b *Research Center for Animal Development Applied Biology, Islamic Azad University of Mashhad Branch, Mashhad, Iran.*

**Keywords:** Cisplatin, Silver, Nanoparticles, Curcumin, Resistance

## Abstract

Today, drug resistance is one of the major problems in fight against cancer. Therefore, combination of therapeutic strategies was raised to effectively improve disease prognosis. In this regard, silver nanoparticles (AgNPs) are considered significant due to their anticancer properties. This study aimed to return sensitivity to cisplatin to A2780 cisplatin-resistance cell lines in the presence of biogenic synthesis curcumin-coated silver nanoparticles (cAgNPs). Synergic cellular effects of cAgNPs and cisplatin on ovarian carcinoma 2780 resistant to cisplatin cells were assessed using MTT assay, Acridine orange (AO)/propidium iodide (PI), DAPI staining, Annexin V/PI assay, and caspase 3/9 activation assay. Finally, expression of p53 and MMP-9 genes were evaluated using semi-quantitative reverse transcription polymerase chain reaction (RT-PCR). According to the results, 8 µg/mL and 62 µg/mL of cAgNPs and cisplatin led to 50% cell death in 48 h, respectively. Therefore, we combined non-toxic concentration of nanoparticles (1-5 µg/mL) with cisplatin (2.5 µg/mL). Decreased proliferation rate was about 50% for synergic use of cisplatin (2.5 µg/mL) and cAgNPs (2 µg/mL). According to the results, cell death induction significantly increased by AO/PI, DAPI staining and Annexin V/PI assay in the combined group. Moreover, activity of caspase 3/9 significantly increased in the mentioned group. The combined use of cAgNPs and cisplatin resulted in upregulated expression of p53 gene and downregulated expression of MPP-9 gene. As observed in this study, a combination of cAgNPs and cisplatin increased the efficiency of apoptosis induction in A2780 cells, compared to the independent use of cisplatin or cAgNPs.

## Introduction

Currently, cancer is one of the major problems of public health. Several methods, such as surgery, radiation, and chemotherapy, have been used to treat cancer. In addition, combination of these strategies has been applied to effectively improve disease prognosis. Given the gradual resistance of many cancers to chemotherapy drugs, which leads to the failure of many forms of treatment methods, the evolution of adaptive mechanisms provides cancer cells with the ability to evade apoptotic execution and bestow upon them a survival advantage. Therefore, it is essential to discover improving therapeutic methods with high efficacy and selectivity to overcome drug resistance. Cisplatin is an anticancer chemotherapeutic drug, which efficiency inhibits the growth of tumor cells (1). This drug crosslinks to DNA and forms DNA adducts, which leads to the interfering with DNA replication and transcription, activating cellular apoptosis in return (2). Initial response of cancer cells to cisplatin-based chemotherapy is usually great; however, there is a delayed relapse due to the development of cisplatin resistance (2). The nanotechnology areas are primarily described with creative materials containing unique properties, raised from their nanoscale size. Today, application of metallic nanoparticles is expanding due to the diverse range of their potential applicability in various fields (3). One of the current applications of nanotechnology is the design of nano-sized materials in order to overcome drug resistance. Several studies have reported the cytotoxicity activity of curcumin-coated silver nanoparticles (cAgNPs) against cancer cells. Silver binds to the cell membrane surface protective SH group, leading to morphological changes in plasma membrane permeability. In addition, this substance causes cell death through affecting the respiratory chain. Therefore, cancer cells are more susceptible to silver nanoparticles due to their higher metabolic rates, compared to normal cells. Silver nanoparticles can be synthesized using chemical and physical methods (4). The green synthesis of nanoparticles offers numerous benefits of eco-friendliness and compatibility for pharmaceutical and biomedical applications since they use no toxic chemicals in the synthesis protocols. Plants, fungi, and microbes are applied for nanoparticle fabrication. Use of the plant extracts for the synthesis of nanoparticles is associated with some advantages, such as cost-effectiveness and eco-friendliness, and the ability to be carried out in only one setup. Previous research reported biosynthesis of silver nanoparticles using extracts of plants, including *Saliva officinalis*, *Achillea biebersteinii* and olive leaf (4-6). Curcumin is a polyphenol, extracted from turmeric spice (Curcuma longa). Many clinical trials have demonstrated the efficacy, pharmacokinetics, and safety of this natural product against numerous human diseases (5). Curcumin inhibits cancer development at cells mutation, metastasis, and proliferation stages without affecting normal cells. In addition, this compound can kill many different types of cancer cells by triggering apoptosis. Given the mentioned benefits, curcumin has been the subject of cancer research for many decades. With this background in mind, this study aimed to evaluate the drug resistance of cisplatin-resistant cells using cAgNPs synthesis as a potential alternative resistance to cisplatin. 

## Experimental

Reagents and media: Curcumin**, **Silver nitrate (AgNO3), MTT [3-(4,5-dimethylthiazol-2-yl)-2,5-diphenyltetrazolium bromide], Acridine orange, propidium iodide, and DAPI (4›, 6-diamidino-2-phenylindole) were obtained from Sigma-Aldrich (Poole, United Kingdom). Fetal bovine serum (FBS) and RPMI-1640 medium were purchased from Invitrogen. The High Pure RNA Isolation Kit and cDNA Synthesis Kit were also purchased from Roche (Mannheim, Germany) and Fermentas Inc. (Vilnius, Lithuania), respectively. In addition, the primers were obtained from Bioneer (Daejeon, Korea), and the commercial cisplatin was purchased from a pharmacy. Annexin V/PI and Caspase Activity Assay Kit were purchased from Abcam Company (Germany). Moreover, A2780 was obtained from Pastor Institute (Iran, Tehran). All the solutions were prepared with double distilled water and other reagents were of analytical grade.

*Synthesis and Characterization of cAgNPs*

CAgNPs was synthesized using curcumin as a reducer agent, and characterized using UV-visible spectrum, Fourier transform infrared (FTIR), and transmission electron microscopy (TEM). This biosynthesized cAgNPs was used for other analyses.

Cell culture and treatment: A2780 cells were cultured in Roswell Park Memorial Institute medium (RPMI) with 10% FBS and 1% penicillin-streptomycin, followed by their incubation at 37 °C in 5% humidified CO2. When reached 90% confluence, the cells were used for other analyses. A2780 was treated with cAgNPs (1, 2, 4, 8 and 16 µg/mL), and cisplatin (5, 10, 15, 30, 60 and 120 µg/mL) for 48 h. After the calculation of IC_50_, the concentration below IC_50_ (2.5 µg/mL of cisplatin and 2 µg/mL CAgNPs) was used for the combined groups, followed by other analyses for these groups.

*Evaluation of cytotoxicity*

The cells were seeded and treated as describe after 48 h. Afterwards, 50 µL MTT solution (5 mg/mL) was added to each well and incubated for two hours in 37 °C. Following that, 100 µL dimethyl sulfoxide (DMSO) was added to solve formazan crystals. The absorbance was read at 570 nm using plate reader and spectrophotometer. The cell viability was calculated using the following equation: 

cell viability (%) = (Atreated/Acontrol) 100, where A treated and A control are the absorbance of the treated and untreated cells, respectively (9). 

*Apoptosis induction assay*

AO/PI staining: the cells were seeded and treated. Forty-eight hours after the seeding, AO/PI staining 10 µL of the live cell sample and 10 µL of AO/PI staining solution were combined and analyzed under the fluorescent microscopy.

DAPI staining: About 5000 cells were cultured in plate containing gelatin-coated coverslips. After 24 h, the culture medium was removed from each well, and the cells were washed with PBS twice. In the next stage, the cells were treated with cAgNPs (2 µg/mL), cisplatin (2.5 µg/mL), or a combination of cAgNPs and cisplatin for 48 h. Afterwards, the cells were fixed with methanol and were stained with DAPI solution (1 mg/mL) for 10–60 sec. Following that, the wells were washed with cold PBS twice. Finally, the comounds were observed under fluorescent microscopy.

*Annexin V/PI staining for apoptosis detection*

 Percentage of early and late apoptotic cells induced with cAgNPs, cisplatin, and a combination of both on A2780 cell were determined by Annexin-V-FITC/PI staining. According to the manufacturer’s instructions, the cells were treated with cAgNPs, cisplatin, or both for 48 h. Afterwards, the cells were harvested and centrifuged at 200×g and suspended in appropriate buffer. Following that, 5 µL Annexin-V-FITC labeling and 5 µL PI solutions were added to the mixture, which were then incubated for five minutes at 25 °C and analyzed with flow cytometry (Bd, UK).

*Caspase 3/9 activation assay*

Caspase 3/9 activities were assessed using the Colorimetric Protease Assay Kit according to the protocol of manufacturer. Briefly, the cells were treated with AgNPs. Apoptosis was induced in the cells treated with cAgNPs and cisplatin for 24 h. After that, the 1-5 × 10^6^ cells were pelleted and re-suspended in 50 μL of chilled cell lysis buffer before centrifuging for one minute. The protein concentration was assayed using the Biuret method. For each assay, 100 μg proteins were diluted with 50 μL cell lysis buffer. Finally, the DEVD-p-NA substrate was added and the samples were read at 400 or 405 nm using a microtiter plate reader (Epoch, US). The fold-increase in caspase-3 activity was determined through comparison with the control groups.

*Semi-quantitative analysis of p53 AND MMP-9 genes expression*

 Changes in the expression of MMP genes were assessed using semi-quantitative reverse transcription polymerase chain reaction (RT-PCR). A2780 cells were treated (according to previous descriptions) for 48 h, and total RNA was extracted (Rouch, Germany). cDNAs were synthesized by reverse transcriptase and amplified by polymerase chain reaction (PCR) with specific primer pairs, as observed in Table 1, using Fermentas PCR Kit (Fermentas, US). The PCR products were analyzed by 1.5% agarose gel electrophoresis, and the gels were observed using gel documentation (UV TEC Cambrege, UK). The tests were repeated in triplicate, and the relative band densities of cDNA.

*Statistical analysis*

Statistical evaluation of the data was performed using one-way analysis of ANOVA, Tukey test. The results were shown as mean ± SD and *p* < 0.05 was calculated as the minimum level of significance.

## Results

**Figure 1:**
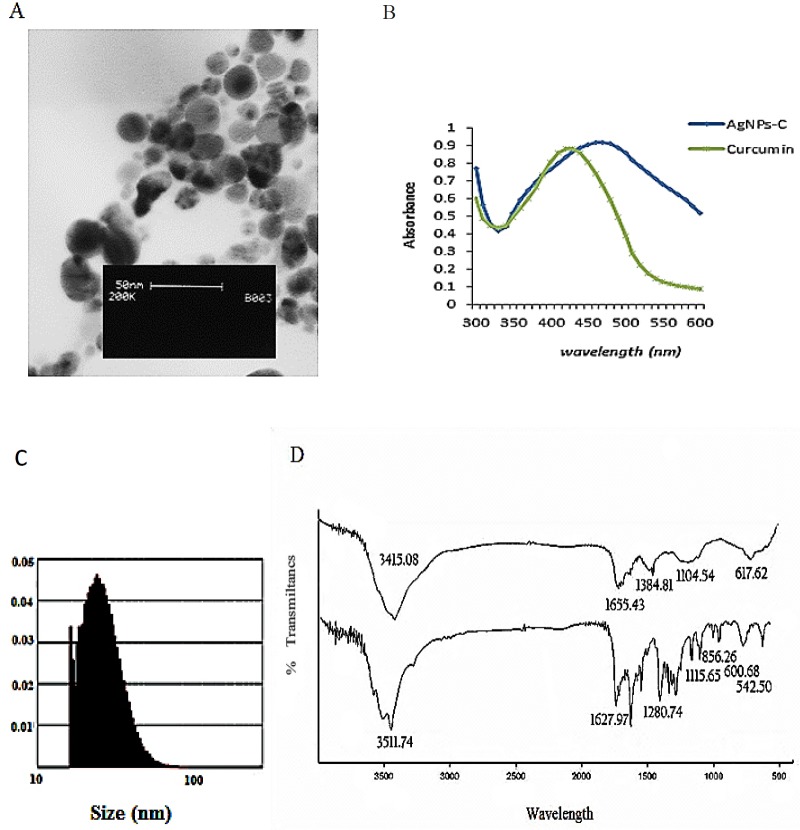
(A) TEM image of AgNPs-C, (B) Uv- visible spectrom from solution contains AgNO3 and curcumin after passing 24 h,

Synthesis and characterization of cAgNPs: In this study, we reported green synthesis of cAgNPs using an average size of 38 ± 2 nm of curcumin and a sharp peak in 450 nm in UV-visible spectrum. FTIR result indicated the capping of nanoparticles by curcumin (Figure 1). These cAgNPs were used to return cisplatin sensitivity to A2780 resistant cells.

**Figure 2:**
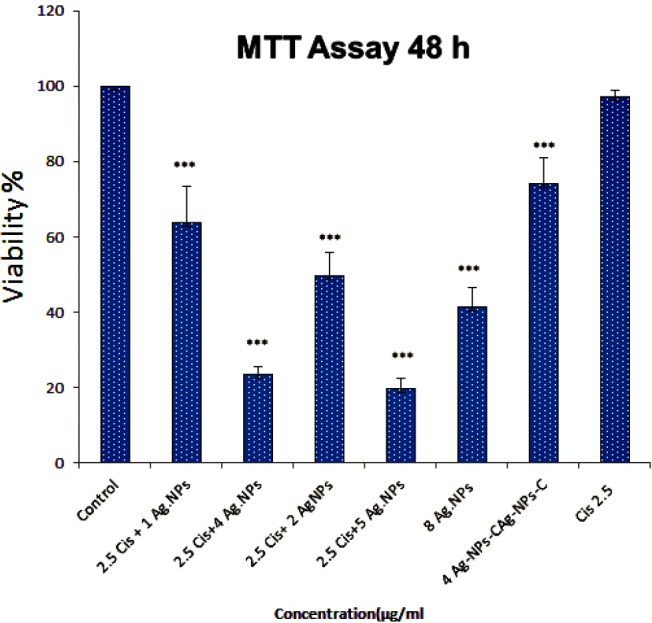
Cell viability was examined by MTT assay. Ag-NPs-C potentiate cisplatin-induced cytotoxicity in A2780 resistant cells. Cells were pretreated with or without Ag-NPs-C and then cultured in the presence or absence of cisplatin for 48 h

Cytotoxicity study: IC_50_ of one of the cAgNPs and cisplatin was examined to assess the efficiency of the combination of these compounds. The obtained results demonstrated that cisplatin and cAgNPs had antiproliferative effects against A2780 resistant cells. Moreover, cisplatin and cAgNPs dose dependent suppressed viability of A2780 cells. As it was expected, there was a significantly higher resistant to cisplatin by A2780 resistant cells, compared to cAgNPs. It is noteworthy that the selected concentrations of cisplatin and cAgNPs were lower than the IC_50_ of A2780 cells. As shown in Figure 1, IC_50_ value for cAgNPs and cisplatin were 8 µg/ mL and 62 µg/mL, respectively. Therefore, concentrations below IC_50_ were selected as the combined doses. According to the results, no significant effect was applied by the concentration of 2.5 µg/mL of cisplatin on death of A2780 cells. Furthermore, combination of 2.5 µg/mL of cisplatin with the selected concentrations of cAgNPs (1, 2, 4 and 5 µg/mL) led to less than 50% cell death. The results indicated that the combined dosage of cAgNPs and cisplatin significantly decreased cell viability of A2780 resistant cells. In this study, the combination ratio of cAgNPs and cisplatin (1:2, 2:2.5, 4:2.5 and 5: 2.5 µg/mL) was associated with cell viability decreased to 63%, 49%, 23%, and 19%, respectively (Figure 2).

**Table 1:** Primer sequence

**Gene**	**Forward primer**	**Reverse primers**
Beta Actin	5ꞌCCC GCC GCCAGC TCA CCA TGG 3ꞌ	5ꞌAAG GTC TCA AAC ATG ATCTGG GTC 3ꞌ
MMP-9	5′CAACATCACCTATTGGATCC 3′	5′CGGGTGTAGAGTCTCTCGCT 3′
p53	5ꞌTTG CCG TCCCAA GCA ATG GA 3ꞌ	5ꞌTCTGGGAAGGGACAGAAGATGAC 3ꞌ

(C) particle size disruption of AgNPs-C, (D) comparing FTIR spectra from AgNPs-C (A) and pure corcumin (B) theses spectra are very similar which indicated that curcumin coated the surface of silver nanoparticles.

**Figure 3:**
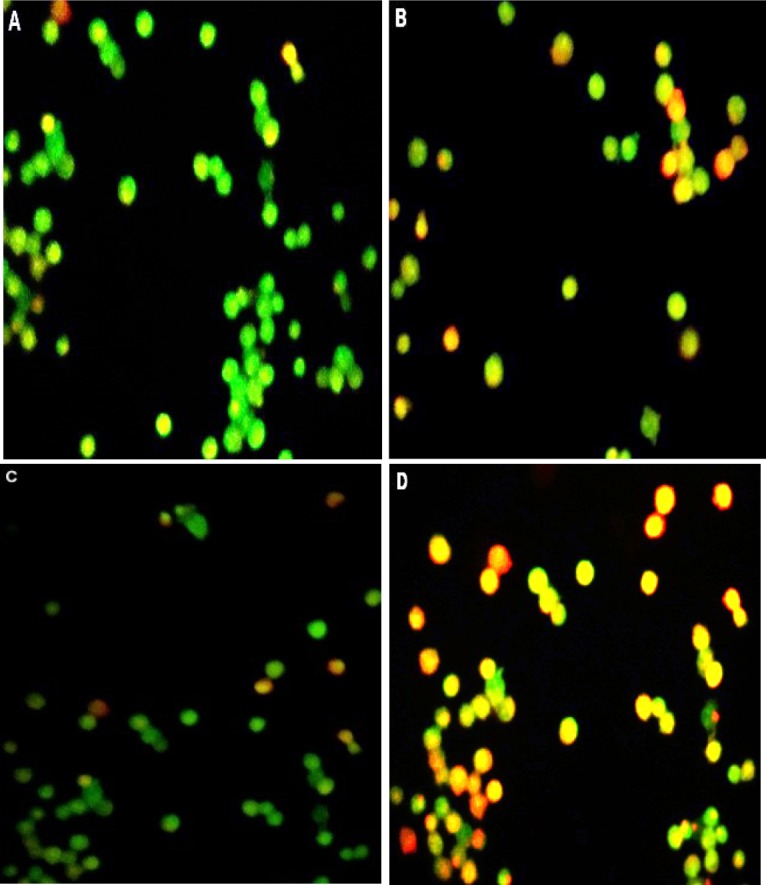
Image of AO/PI double-stained A2780 cells. (A) Control group was seen uniformly green. (B) Cell exposer to Ag-NPs, Early apoptosis features were observed orange (green) amongst the fragmented DNA. (C) Cells treated with cisplatine red cell were necrotic cells. (D) Cells treated with both Ag-NPs-C and cisplatin

**Figure 4:**
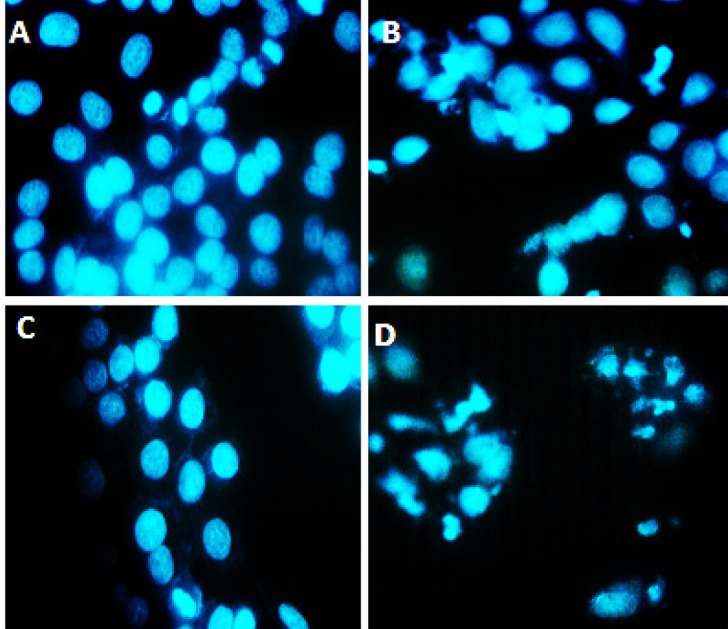
DAPI nuclear staining, (A) Represents control group that did not receive any treatment and cell nuclei are intact, (B) Represents the group that treated with only silver nano particles in this group some nucleus of cells are fracture, (C) Cells treated with cisplatin. The most cell nuclei are intact, (D) Cells treated with cisplatin and silver nanoparticles together. In this group cell nucleus are fractures and showed chromatin condensation (arrow), the apoptotic features in synergistic groups was higher compared to groups that were treated with cisplatin or silver nanoparticles alone were used

**Figure 5:**
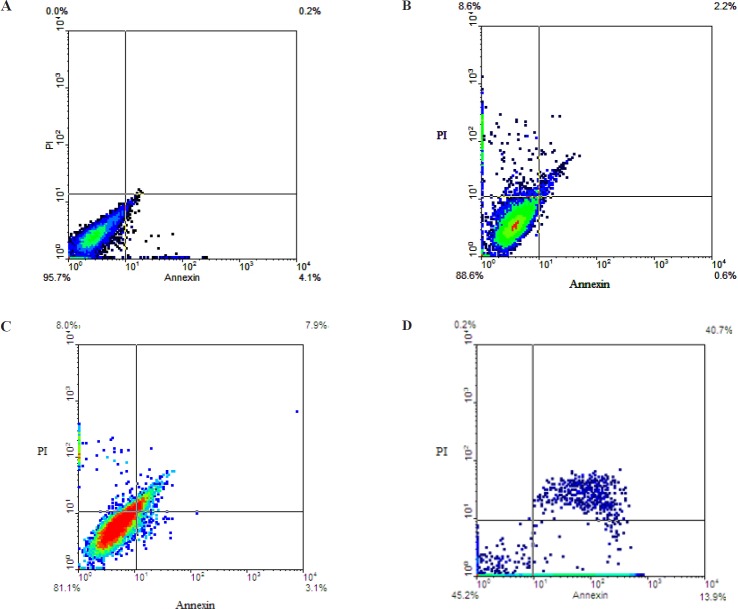
Comparing apoptosis induction through cisplatin (2.5 μg/mL), Ag-NPs (2 μg/mL) and both of Ag-NPs-C and cisplatin

(cisplatin + Ag-NPs-C) at 48 h.

**Figure 6:**
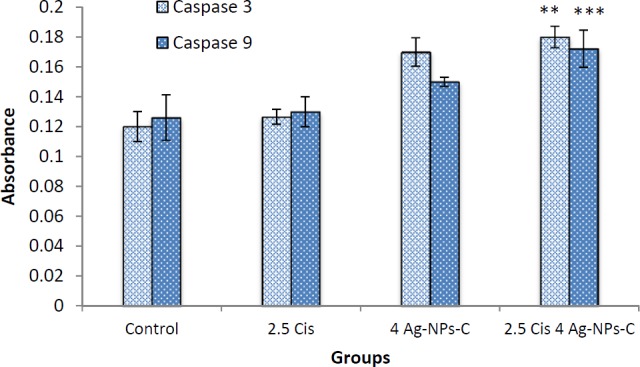
The activity of caspases -3/9 was increased after treatment, which indicates that apoptosis is significant (*p* > 0.05), Data are presented as mean ± SD

**Figure 7:**
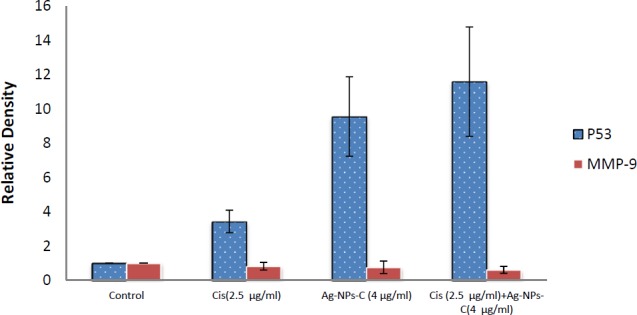
Scanning densitometry of semi quantitative RT-PCR products for p53 gene, the control group was assumed to represent one number greater than represent more expression compared control group (p53). Data are presented as means ± SD

*Apoptosis induction assay*

AO/EB staining: Analysis of A2780 cells after 48 h of treatment with independent use of two µg/mL of cAgNPs and 2.5 µg/mL of cisplatin and mixture of these concentrations through AO/EB staining are presented in Figures 3A-3D. According to this figure, green live A2780 cells with a normal morphology were observed in the control group. In contrast, early apoptotic cells with yellow green stain and late apoptotic cells with red stain in A2780 cells were seen in the test groups. In addition, early apoptotic cells with bright green nucleus, nuclear migration, and chromatin coagulation were observed in the treated group. The results suggested that while only slight changes were observed in the cells treated with an independent dose of cAgNPs or cisplatin at the same concentrations, the combined mixture of cAgNPs and cisplatin revealed more apoptotic activity in A2780 cells. However, early apoptotic (yellow-green) and late apoptotic (red) cells were increased in combined groups, compared to the independent use of cisplatin or cAgNPs. In this regard, our findings are in line with previously mentioned MTT assay studies.

DAPI staining: The most popular nuclear stain for identification of nuclei morphological change during apoptosis is DAPI. This dye specifically stains nuclei. Cell permeability to DAPI increased during the apoptosis process; therefore, high blue fluorescence exited in apoptotic cells, compared to the normal cells. Moreover, we were able to analyze morphological changes in apoptosis characteristics, including nuclear fragmentation, through nuclear staining. In this study, DAPI staining was used to compare the cell nucleus changes in the different treatment groups. According to the results of DAPI staining, higher levels of DNA fragmentation and condensed chromosome were observed in the combined groups, compared to the groups treated an independent dose of cAgNPs or cisplatin. However, for normal cells, round nucleus was stained uniformity and its margin was clear (Figures 4A-4D). With respect to this notion, this type of cell is resistant to cisplatin.

Annexin V/PI staining for apoptosis detection: Percentage of apoptosis rate was determined using an Annexin V-FITC Apoptosis Detection Kit. Annexin V-FITC to label cell surface inverted phosphatidylserine was used on apoptotic cells and PI was applied for detected necrotic cells. Positioning of quadrants on Annexin V/PI dot plots was performed, followed by the discrimination of living cells (Annexin V−/PI−), primary/early apoptotic cells (Annexin V+/PI−), late apoptotic/secondary apoptotic cells (Annexin V+/PI+) and necrotic cells (Annexin V−/PI+). Moreover, A2780 cells were treated with independent doses of 2.5 µg/mL cisplatin and 2 µg/mL of cAgNPs and a mixed dose of 2.5 of µg/mL cisplatin + 2 µg/mL of cAgNPs (Figures 5A-5D). According to the results, 2.5 µg/mL of cisplatin and 2 µg/mL of cAgNPs led to the death of 11.4% and 19% of treated cells, respectively. As presented in the following figure, the percentages of early (13.9%) and late apoptotic cell (45.2) increased in combined groups, compared to the independent use of these compounds. It could be concluded that cisplatin increased the cytotoxicity of cAgNPs.

Caspase 3/9 activation assay: The cells were treated with independent doses of cisplatin (2.5 µg/mL) and cAgNPs (2 µg/mL) and a mixed dose of 2.5 of µg/mL cisplatin + 4 µg/mL of cAgNPs for 48 h. As shown in Figure 6, combination of cAgNPs and cisplatin resulted in an increased activation of caspase-3 and caspase-9, compared to the independent use of these compounds. the Increased caspase activation refers to an increase in apoptosis in treated cells using both cisplatin and cAgNPs. This result is consistent with the data from AO/EB, DAPI staining, indicating that the apoptosis induction was more efficiency in groups treated with a mixture of cisplatin and cAgNPs in A2780 cells. 

Semi-quantitative analysis of p53 and MMP-9 genes expression: As observed in Figure 7, p53 expression was significant increased when a mixed dose of cisplatin and cAgNPs was used, compared to the independent use of these compounds. According to the results, p53 expression was upregulated in the presence of cisplatin. Therefore, it could be suggested that silver nanoparticles and cisplatin had synergistic effects and could enhance the performance of each other and return p53 functions in order to sensitize A2780 cells to apoptosis. In this study, anti-metastatic potential of cAgNPs and cisplatin was evaluated through analyzing the changes in MMP-9 gene expression. The results indicated that the expression of pro-metastatic MMP-9 was efficiency inhibited in combined groups. Therefore, it could be concluded that the simultaneous application of silver nanoparticles and cisplatin efficiently downregulated MMP-9 expression, which resulted in the reduced metastatic potential of A2780 cancer cells.

## Discussion

A hallmark of all types of human cancers is acquired drug resistance. Apoptosis resistance may contribute to drug resistance since the majority of current anticancer studies have tried to activate cell death pathways, including apoptosis, in cancer cells (9, 10). Modulation of apoptosis may influence resistance to chemotherapy and, consequently, affect the outcome of cancer treatment. Ovarian cancer, one of the most important causes of mortality in women, is often associated with drug resistance (11, 12). The present study was conducted to overcome A2780 cellular resistance to cisplatin, silver nanoparticles or a combination of these compounds. We aimed to report green synthesis of AgNPs using pure curcumin, which plays roles both as capping and reducing agents. According to the results of the current research, cAgNPs coated with curcumin and silver nanoparticles acted as carrier for curcumin delivery. In addition, minimum toxic concentrations of cAgNPs and cisplatin were used to assess the efficiency of a combination of these compounds in cytotoxicity assay. According to the results of cytotoxicity assay, the cytotoxic effect of cisplatin on A2780 resistant cells increased in the presence of cAgNPs. The IC_50_ value decreased to 2.5 µg/mL in cells treated with cisplatin in the presence of cAgNPs, whereas this value was 62 µg/mL in cells treated with an independent dose of cisplatin. Efficiency of apoptosis induction through cAgNPs, cisplatin alone, and synergic use of both was exanimated by various assay. 

In addition, AC/EB and DAPI staining were used to obtain more information regarding morphological changes of cells. The results indicated a significant morphological change in cells treated with a combined dose of cAgNPs and cisplatin, compared to the independent use of the mentioned compounds. Furthermore, the results of Annexin V/PI demonstrated a greater apoptosis-inducing effect of cisplatin on A2780 resistant cells when combined with cAgNPs, compared to the independent use of cisplatin or cAgNPs. There are well-characterized caspase activation pathways mediating apoptosis. Caspase-9 activates disassembly in response to agent, and caspase-3 by proteolytic cleavage, leading to the vital cleave of the cellular proteins or other caspases by caspase-3 (13, 14). 

Caspase 3/9 activations were evaluated to compare apoptosis induction in the treated cells with cAgNPs, cisplatin, and cAgNPs+ cisplatin. The obtained results indicated that combination of cAgNPs with cisplatin resulted in much enhanced caspase-3 and caspase-9 activations. Moreover, cisplatin-induced apoptosis increased in the presence of silver nanoparticles. In this regard, Hekmat *et al.* (2012) reported that synergic use of doxorubicin and AgNPs cooperatively destroyed more T47D and MCF7 cancer cells (12). In another study by Xiong *et al. *(2015), it was demonstrated that gold nanoparticles prevented cisplatin-induced acquired chemoresistance and stemness in ovarian cancer cells and sensitize them to cisplatin. According to the results of the mentioned study, gold nanoparticles mechanistically (AuNPs) prevented cisplatin-induced activation of Akt and NF-κB signaling axis in ovarian cancer cells, which are critical for EMT, stem cell maintenance and drug resistance (13). The p53 expression was analyzed in treated and untreated cells to evaluate the molecular mechanism that sentisized A2780 cells to cisplatin in the presence of silver nanoparticles. p53 is one of the most significantly studied tumor suppressor proteins and a reliable DNA repair before DNA replication (14). Generally, p53 gene mutation leads to the loss of wild-type p53 activity and frequent cause of abnormalities in different tumor types (15). In addition, p53 function loss is one of the most predominant mechanisms of resistance (14). *In-vitro* and *in-vivo* study have indicated the return of p53 gene activity in some cancer cells, which leads to apoptosis induction (16). It has been reported that high levels of p53 expression and DNA-damaging agents, such as cisplatin and radiation, work synergistically to induce apoptosis and reverse the resistance in cancer cells (6, 7). Literature review indicated a significant increase in p53 expression in synergic groups. Therefore, it could be concluded that silver nanoparticles and cisplatin had synergistic effects and enhanced the performance of each other in order to return the function of p53 to sentisize A2780 cells to apoptosis. MMP-9 is one of the most important proteins involved in cancer cell metastasis. Studies have shown an overexpressed status of MMP-9 in various cancer cells (19). In the current research, the expression of MMP-9 was down regulated in the cells treated with a combined dose of cisplatin + silver nanoparticles. Therefore, it seems that simultaneous application of cAgNPs and cisplatin efficiently downregulated MMP-9 expression, which reduced the metastatic potential of A2780 cancer cells. There is a speculation over the possible mechanisms of sensitive A2780 cells resistance; in this regard, Sun *et al.* 2016 marked that short-term exposure to AgNPs at low doses inducing a substantial increase in cell permeability. According to the results of the mentioned research, this effect is independent of oxidative stress and apoptosis (20). It could be argued that low doses of AgNPs interacted with cell membrane and increased cell permeability. This way, accumulation of cisplatin subsequently in the cells and its sensitivity increased. In addition, it was demonstrated that AgNPs increased the concentration of super oxides in living cells, causing genotoxic phenomenon and changes in cell permeability (18-20), which might have increased the sensitivity of A2780 cells to cisplatin. In fact, DNA damage stimulated the production of the Reactive oxygen species (ROS), leading to the activation of p53. In return, the activity of p53 increased the sensitivity of cancer cells. As confirmed by previous research, loss of p53 activity is associated with cisplatin resistance (22). In the present study, pure curcumin was used to synthesize silver nanoparticles. According to the results, curcumin sensitized resistant cells toward cisplatin-induced death (23). In general, this compound binds to the surface of silver nanoparticles (As evidence from FTIR study), which may also be involved in the process of increasing sensitivity to cisplatin. However, further studies should be conducted to confirm these data. In the current research, the novel strategy to potentiate cisplatin-induced apoptosis in cancer cells by cAgNP was first introduced. Our findings indicated that application of cAgNPs as a chemo sensitizer of cisplatin might represent less side effects and more efficiency when combined with cisplatin.
